# Prediction of Masked Hypertension and Masked Uncontrolled Hypertension Using Machine Learning

**DOI:** 10.3389/fcvm.2021.778306

**Published:** 2021-11-19

**Authors:** Ming-Hui Hung, Ling-Chieh Shih, Yu-Ching Wang, Hsin-Bang Leu, Po-Hsun Huang, Tao-Cheng Wu, Shing-Jong Lin, Wen-Harn Pan, Jaw-Wen Chen, Chin-Chou Huang

**Affiliations:** ^1^School of Medicine, College of Medicine, National Yang Ming Chiao Tung University, Taipei, Taiwan; ^2^Division of Cardiology, Department of Medicine, Taipei Veterans General Hospital, Taipei, Taiwan; ^3^Cardiovascular Research Center, National Yang Ming Chiao Tung University, Taipei, Taiwan; ^4^Healthcare and Management Center, Taipei Veterans General Hospital, Taipei, Taiwan; ^5^Department of Critical Care Medicine, Taipei Veterans General Hospital, Taipei, Taiwan; ^6^Institute of Clinical Medicine, National Yang Ming Chiao Tung University, Taipei, Taiwan; ^7^Taipei Heart Institute, Taipei Medical University, Taipei, Taiwan; ^8^Institute of Biomedical Sciences, Academia Sinica, Taipei, Taiwan; ^9^School of Medicine, Institute of Pharmacology, National Yang Ming Chiao Tung University, Taipei, Taiwan

**Keywords:** ambulatory blood pressure monitoring, artificial intelligence, machine learning, hypertension, masked hypertension, masked uncontrolled hypertension

## Abstract

**Objective:** This study aimed to develop machine learning-based prediction models to predict masked hypertension and masked uncontrolled hypertension using the clinical characteristics of patients at a single outpatient visit.

**Methods:** Data were derived from two cohorts in Taiwan. The first cohort included 970 hypertensive patients recruited from six medical centers between 2004 and 2005, which were split into a training set (*n* = 679), a validation set (*n* = 146), and a test set (*n* = 145) for model development and internal validation. The second cohort included 416 hypertensive patients recruited from a single medical center between 2012 and 2020, which was used for external validation. We used 33 clinical characteristics as candidate variables to develop models based on logistic regression (LR), random forest (RF), eXtreme Gradient Boosting (XGboost), and artificial neural network (ANN).

**Results:** The four models featured high sensitivity and high negative predictive value (NPV) in internal validation (sensitivity = 0.914–1.000; NPV = 0.853–1.000) and external validation (sensitivity = 0.950–1.000; NPV = 0.875–1.000). The RF, XGboost, and ANN models showed much higher area under the receiver operating characteristic curve (AUC) (0.799–0.851 in internal validation, 0.672–0.837 in external validation) than the LR model. Among the models, the RF model, composed of 6 predictor variables, had the best overall performance in both internal and external validation (AUC = 0.851 and 0.837; sensitivity = 1.000 and 1.000; specificity = 0.609 and 0.580; NPV = 1.000 and 1.000; accuracy = 0.766 and 0.721, respectively).

**Conclusion:** An effective machine learning-based predictive model that requires data from a single clinic visit may help to identify masked hypertension and masked uncontrolled hypertension.

## Introduction

Hypertension is a major global health risk, affecting 1.13 billion people worldwide (1). However, almost half of people are unaware that they have hypertension (2). It is therefore important to improve the diagnosis and monitoring of hypertension for better management of blood pressure (BP) and to reduce the risk of developing future cardiovascular diseases (CVD) (3).

Masked hypertension (MH) or masked uncontrolled hypertension (MUCH) is defined as normotensive office BP and hypertensive out-of-office BP (4–7). MH refers to treatment-naïve patients and MUCH to patients with prior hypertension treatments. International registries show that MH/MUCH is a highly prevalent condition, present in up to one in three office-controlled patients (8). Patients with MH/MUCH have an increased risk of mortality and cardiovascular events (6, 9, 10).

Currently, the diagnosis of MH/MUCH depends on out-of-office BP measurement, including ambulatory BP monitoring (ABPM) and home BP monitoring (HBPM) (4–7), which take at least 24 h or 7 days, respectively. Whether MH/MUCH patients can be diagnosed early based on the clinical features of a single outpatient visit is still an open question.

Artificial intelligence (AI) approaches have revolutionized the way data can be processed and analyzed. Several studies have shown the potential benefits of AI in the prediction of cardiac arrhythmias, coronary artery disease, heart failure, and stroke (11, 12). However, the application of AI in hypertension diagnosis or classification is still limited (13).

The current study aimed to develop machine learning-based prediction models using accessible clinical characteristics as input features to identify patients with MH/MUCH in actual clinical settings. The models we developed may facilitate the diagnosis of MH/MUCH.

## Materials and Methods

### Data Sources and Patient Selection

Data for this study were derived from two cohorts. In the first cohort (cohort 1), patients with hypertension were recruited from six medical centers in Taiwan between 2004 and 2005. The inclusion criteria were as follows: age 20–50 years; patients with essential hypertension; body mass index (BMI) ≤ 35 kg/m^2^; fasting glucose level <126 mg/dL without diabetes mellitus; no medical history of severe diseases, including malignancy or failure of the heart, lungs, kidneys, or liver; and no acute disease within 2 weeks prior to the visit. Patients with secondary hypertension were excluded from the study. The inclusion and exclusion criteria were described in detailed in a previous unrelated study (14). The study protocol was approved by the ethics committees of Academia Sinica and the six medical centers.

In the second cohort (cohort 2, the external validation set), patients with hypertension who visited the outpatient clinic of Taipei Veteran General Hospital between 2012 and 2020 were included. The inclusion criteria were as follows: age ≥20 years; patients with essential hypertension; without a medical history of severe diseases, including malignancy or failure of the heart, lungs, kidneys, or liver; and no acute disease within 2 weeks prior to the visit. Patients with secondary hypertension were excluded from the study. The inclusion and exclusion criteria were described in detail in a previous unrelated study (15).

All patients in the two cohorts agreed to participate and signed the informed consent document for the study. Data collection from both cohorts were conducted in accordance with the principles of the Declaration of Helsinki.

### Study Design

**Figure 1:**
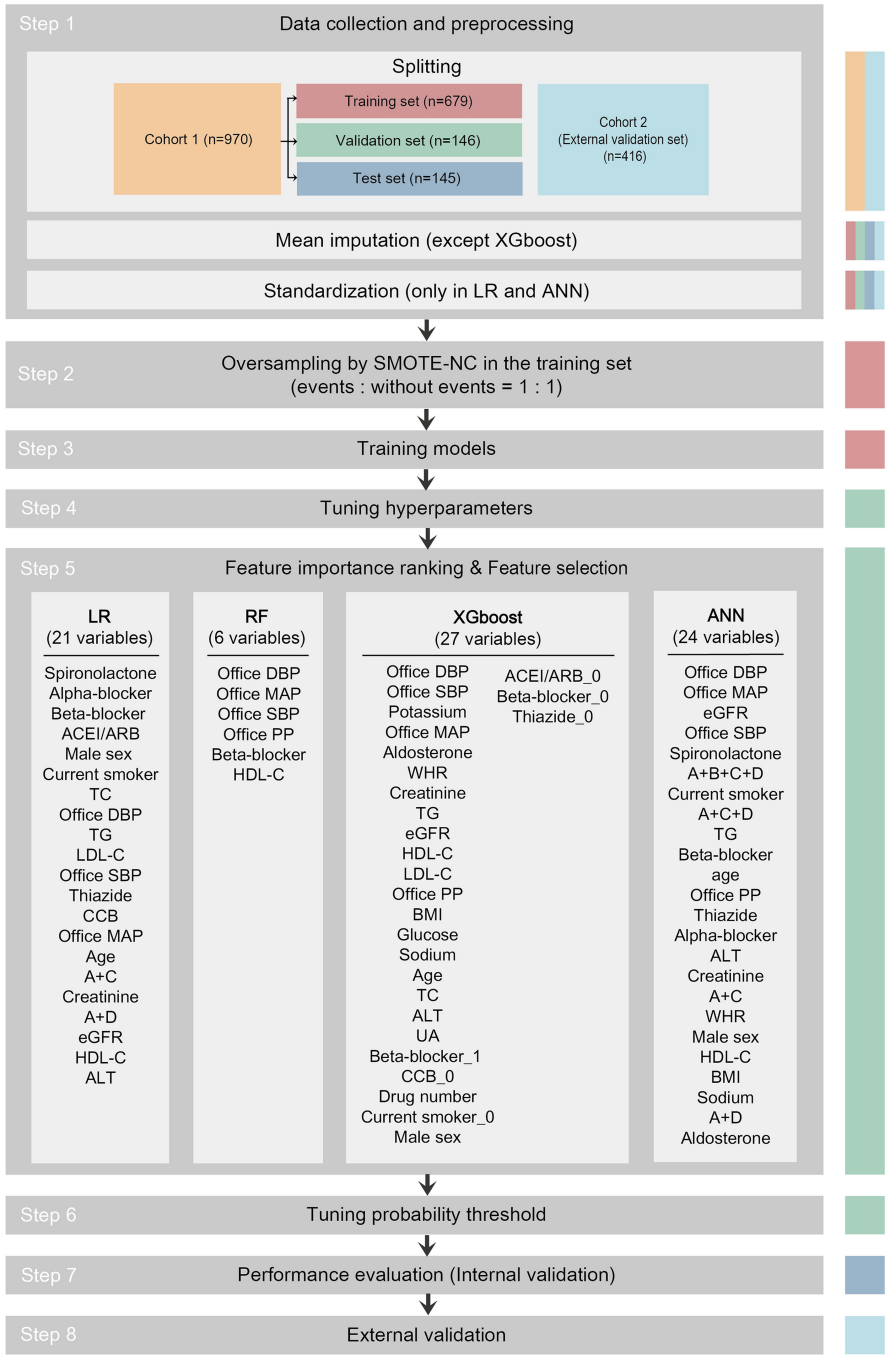
Study flowchart. The data set used in each step is indicated as colored columns on the right side (training set for step 2 and 3, validation set for step 4 to 6, test set for step 7, cohort 2 for step 8). Mean imputation was done to all data sets using the mean of the training set in the LR, RF, and ANN models after splitting. Standardization was done to all data sets in the LR and ANN models after mean imputation. In step 5, 21 among 33 candidate variables were selected as predictor variables in the LR model, 6 in RF, 27 in XGboost, and 24 in ANN. The predictor variables were written in descending order of importance. ACEI/ARB, angiotensin-converting enzyme inhibitor/angiotensin receptor blocker; ACEI/ARB_0, dummy variable of not taking ACEI/ARB; A+B+C+D, combination of ACEI/ARB and beta-blocker and CCB and thiazide; A+C, combination of ACEI/ARB and CCB; A+C+D, combination of ACEI/ARB and CCB and thiazide; A+D, combination of ACEI/ARB and thiazide; ALT, alanine aminotransferase; ANN, artificial neural network; Beta-blocker_1, dummy variable of taking beta-blocker; Beta-blocker_0, dummy variable of not taking beta-blocker; BMI, body mass index; CCB, calcium channel blocker; CCB_0, dummy variable of not taking CCB; Current smoker_0, dummy variable of not current smoker; DBP, diastolic blood pressure; eGFR, estimated glomerular filtration rate; HDL-C, high-density lipoprotein-cholesterol; LDL-C, low-density lipoprotein-cholesterol; LR, logistic regression; MAP, mean arterial pressure; PP, pulse pressure; SMOTE-NC, synthetic minority oversampling technique–nominal continuous; Thiazide_0, dummy variable of not taking thiazide; RF, random forest; SBP, systolic blood pressure; TC, total cholesterol; TG, triglyceride; UA, uric acid; WHR, waist-to-hip ratio; XGboost, eXtreme Gradient Boosting.

Data from cohort 1 were used to develop prediction models to identify patients with MH/MUCH and for internal validation. Data from cohort 2 were used for external validation. The study flowchart is shown in Figure 1.

### Data Collection and Candidate Variables

In total, 73 and 53 variables were recorded in cohort 1 and 2, respectively (Supplementary Table 1). Of these, 33 variables were selected or derived as the candidate variables for the model based on previous literature and taking into account the accessibility of data from outpatient clinics (4, 5, 7, 16–27). These candidate variables were as follows: 1. demographic data [age, male sex, BMI, waist-to-hip ratio (WHR), current smoker]; 2. office BP parameters [office systolic BP (SBP), office diastolic BP (DBP), office mean arterial pressure (MAP), office pulse pressure (PP)]; 3. antihypertensive drug usage [angiotensin-converting enzyme inhibitor/angiotensin receptor blocker (ACEI/ARB), beta-blocker, calcium channel blocker (CCB), thiazide, spironolactone, alpha-blocker, combination of ACEI/ARB and CCB, combination of ACEI/ARB and thiazide, combination of ACEI/ARB and CCB and thiazide, combination of CCB and thiazide, combination of ACEI/ARB and beta-blocker and CCB and thiazide, antihypertensive drug number]; and 4. biochemical profiles [total cholesterol (TC), triglyceride (TG), high-density lipoprotein-cholesterol (HDL-C), low-density lipoprotein-cholesterol (LDL-C), creatinine, sodium, potassium, alanine aminotransferase (ALT), uric acid (UA), glucose, aldosterone, estimated glomerular filtration rate (eGFR)] (Supplementary Material 1).

### Definition of MH/MUCH

MH and MUCH were defined as office BP <140/90 mmHg and 24-h average BP ≥ 130/80 mmHg and/or awake (daytime) BP ≥ 130/80 mmHg and/or asleep (nighttime) BP ≥ 120/70 mmHg in untreated and treated patients, respectively (5–7). MH and MUCH were labeled as events based on the office BP and 24-h ambulatory BP measured in each participant in both cohorts.

### Prediction Models

Logistic regression (LR), random forest (RF), eXtreme Gradient Boosting (XGboost), and artificial neural network (ANN) were used as the classifiers to obtain a comprehensive spectrum of prediction models. All the models were developed using RStudio (version 1.3.1056, RStudio, PBC, Boston, MA, USA). The algorithms and packages used are listed in Supplementary Table 2. All models returned discriminative outputs of 1 to indicate events or 0 to indicate non-events.

### Development of Prediction Models

As shown in Figure 1, the pipeline to develop the prediction models consisted of the following steps: 1. data collection and preprocessing; 2. oversampling; 3. training models; 4. tuning hyperparameters; 5. importance ranking of 33 candidate variables and feature selection for predictor variables; 6. tuning probability threshold; 7. performance evaluation (internal validation); and 8. external validation.

Participants in cohort 1 (*n* = 970) were randomly split into training set (*n* = 679), validation set (*n* = 146), and test set (*n* = 145) in a 0.7/0.15/0.15 ratio with balanced levels. Missing values in the training set, validation set, test set, and external validation set were replaced by the mean of all available values for the same variable in the training set in the LR, RF, and ANN models (only 3 missing data points out of 26,190 data points in cohort 1 and 63 missing data points out of 11,232 data points in cohort 2). In the LR and ANN models, all variables were scaled (normalized) by subtracting the mean and then dividing by the standard deviation (SD) of the training set.

Given that there was a class imbalance between events and non-events and to overcome the accuracy paradox, we performed the synthetic minority oversampling technique–nominal continuous (SMOTE-NC) to equalize the number of events and non-events in the training set (random oversampling and random undersampling were also performed, but with poorer performance) (28).

To obtain the maximum area under the receiver operating characteristic curve (AUC) in the validation set during model training, we tuned the hyperparameters using a random search technique (29). Feature importance ranking and supervised feature selection were performed to prevent overfitting and to achieve the maximum AUC in the validation set (30). The details of feature selection are presented in Supplementary Material 2. We established the confusion matrix and calculated F_1_ score (= $2\times \frac{precision\times recall}{precision+recall}$) while changing the decision threshold of the classifier from 0 to 1 (threshold-moving) in the validation set. We then selected the optimal probability threshold yielding the largest F_1_ score and published the final models (Supplementary Material 3). The test set and the external validation set were always independent of the training and tuning processes during the development of the models.

### Performance Metrics of Internal Validation

To evaluate the performance of various models in the test set, we computed the sensitivity, specificity, positive predictive value (PPV), negative predictive value (NPV), accuracy, and F_1_ score. Receiver operating characteristic (ROC) curves were plotted along with the AUC. For AUC calculation, all predicted results were converted to probabilities.

### External Validation

The external validity of the model was then evaluated with the external validation set. Model discrimination was assessed by plotting ROC curves and calculating the AUC. The sensitivity, specificity, PPV, NPV, accuracy, and F_1_ score were also computed.

### Statistical Analysis

Quantitative variables are expressed as mean ± SD, and categorical variables are expressed as percentages. Continuous parametric data between cohorts 1 and 2 were compared using an unpaired Student's *t*-test. Continuous parametric data between the training set, validation set, and test set were compared by one-way analysis of variance. Non-parametric data were compared using the Mann-Whitney test. Categorical variables were analyzed using the chi-square test or Fisher's exact test. Spearman's rank correlation coefficients were calculated between candidate variables. Statistical significance was inferred at a two-sided *P*-value < 0.05. Statistical analysis was performed using the SPSS software (version 21.0, SPSS Inc., Chicago, IL, USA).

## Results

### Baseline Characteristics

In cohort 1, there were 970 patients with hypertension; their mean age was 41.0 ± 7.2 years and 68.5% of them were male. Heat map showing the Spearman's correlation coefficients with significance levels of pairwise comparison between variables is presented in Supplementary Figure 1. In cohort 2, there were 416 patients with hypertension; their mean age was 62.0 ± 14.2 years and 57.7% of them were male. Compared to cohort 1, cohort 2 patients had higher WHR (*P* < 0.001), office SBP (*P* < 0.001), office PP (*P* < 0.001), but lower office DBP (*P* < 0.001); used more antihypertensive drugs (*P* < 0.001), including more ACEI/ARB (*P* < 0.001), CCB (*P* < 0.001), alpha-blockers (*P* = 0.021), combination of ACEI/ARB and CCB (*P* < 0.001), combination of ACEI/ARB and CCB and thiazide (*P* < 0.001), and combination of ACEI/ARB and beta-blocker and CCB and thiazide (*P* = 0.001), but less beta-blockers (*P* < 0.001); had lower levels of TG (*P* < 0.001), TC (*P* < 0.001), and potassium (*P* < 0.001), as well as lower eGFR (*P* < 0.001) (Table 1). Baseline characteristics of the training set, validation set, and test set were similar, except HDL-C (*P* = 0.038) (Table 2).

**Table 1:** Baseline characteristics and the proportion of MH/MUCH in the two cohorts.

	**Cohort 1 (*n* = 970)**	**Cohort 2 (*n* = 416)**	***P*-value**
**Demographic data**			
Age, years	41.0 ± 7.2	62.0 ± 14.2	<0.001
Male sex, *n* (%)	664 (68.5%)	240 (57.7%)	<0.001
BMI, kg/m2	26.5 ± 3.4	26.1 ± 3.7	0.036
WHR, %	87.6 ± 6.1	91.5 ± 7.1	<0.001
Current smoker, *n* (%)	232 (23.9%)	22 (5.3%)	<0.001
**Office BP parameters**			
Office SBP, mmHg	126.1 ± 14.5	131.6 ± 16.8	<0.001
Office DBP, mmHg	84.9 ± 11.7	81.8 ± 10.5	<0.001
Office MAP, mmHg	98.7 ± 11.9	98.4 ± 11.0	0.671
Office PP, mmHg	41.2 ± 9.8	49.8 ± 14.6	<0.001
**Antihypertensive drug usage**			
Antihypertensive drug number, *n*	1.5 ± 1.0	1.9 ± 0.9	<0.001
ACEI/ARB, *n* (%)	410 (42.3%)	276 (66.3%)	<0.001
Beta-blocker, *n* (%)	437 (45.1%)	104 (25.0%)	<0.001
CCB, *n* (%)	405 (41.8%)	306 (73.6%)	<0.001
Thiazide, *n* (%)	157 (16.2%)	83 (20.0%)	0.089
Spironolactone, *n* (%)	7 (0.7%)	5 (1.2%)	0.276
Alpha-blocker, *n* (%)	22 (2.3%)	19 (4.6%)	0.021
Combination of ACEI/ARB and CCB, *n* (%)	71 (7.3%)	96 (23.1%)	<0.001
Combination of ACEI/ARB and thiazide, *n* (%)	36 (3.7%)	12 (2.9%)	0.440
Combination of CCB and thiazide, *n* (%)	13 (1.3%)	4 (1.0%)	0.557
Combination of ACEI/ARB and CCB and thiazide, *n* (%)	26 (2.7%)	34 (8.2%)	<0.001
Combination of ACEI/ARB and beta-blocker and CCB and thiazide, *n* (%)	13 (1.3%)	17 (4.1%)	0.001
**Biochemical profiles**			
TC, mg/dL	195.8 ± 35.4	184.4 ± 30.9	<0.001
TG, mg/dL	166.1 ± 112.2	130.3 ± 90.0	<0.001
HDL-C, mg/dL	45.4 ± 12.1	48.6 ± 13.0	<0.001
LDL-C, mg/dL	126.0 ± 31.4	112.0 ± 27.1	<0.001
Creatinine, mg/dL	0.8 ± 0.2	0.9 ± 0.2	0.324
eGFR, mL/min/1.73 m2	129.4 ± 38.3	86.1 ± 19.2	<0.001
Sodium, mmol/L	141.3 ± 2.6	141.0 ± 2.5	0.076
Potassium, mmol/L	4.0 ± 0.3	3.9 ± 0.6	<0.001
ALT, U/L	27.6 ± 19.3	26.1 ± 16.6	0.167
UA, mg/dL	6.6 ± 1.7	6.1 ± 1.5	<0.001
Glucose, mg/dL	98.1 ± 9.2	101.9 ± 18.0	<0.001
Aldosterone, pg/mL	226.5 ± 121.0	122.0 ± 11.5	<0.001
**Ambulatory BP parameters**			
24-h SBP, mmHg	123.2 ± 12.2	122.0 ± 11.5	0.068
24-h DBP, mmHg	82.6 ± 9.5	73.2 ± 8.3	<0.001
Daytime SBP, mmHg	126.1 ± 12.7	123.9 ± 11.9	0.002
Daytime DBP, mmHg	84.9 ± 9.9	74.8 ± 8.5	<0.001
Nighttime SBP, mmHg	114.0 ± 12.8	117.6 ± 12.9	<0.001
Nighttime DBP, mmHg	74.7 ± 10.0	69.5 ± 9.3	<0.001
MUCH/MH, *n* (%)	386 (39.8%)	140 (33.7%)	0.031

*ACEI, angiotensin converting enzyme inhibitor; ALT, alanine aminotransferase; ARB, angiotensin receptor blocker; BMI, body mass index; BP, blood pressure; CCB, calcium channel blocker; DBP, diastolic blood pressure; eGFR, estimated glomerular filtration rate; HDL-C, high-density lipoprotein-cholesterol; LDL-C, low-density lipoprotein-cholesterol; MAP, mean arterial pressure; MH, masked hypertension; MUCH, masked uncontrolled hypertension; PP, pulse pressure; SBP, systolic blood pressure; TC, total cholesterol; TG, triglyceride; UA, uric acid; WHR, waist-hip ratio*.

**Table 2:** Baseline characteristics and the proportion of MH/MUCH in the training set, validation set, and test set.

	**Training set** **(*n* = 679)**	**Validation set** **(*n* = 146)**	**Test set** **(*n* = 145)**	***P*-value**
**Demographic data**				
Age, years	41.2 ± 6.8	40.5 ± 8.1	41.1 ± 7.8	0.634
Male sex, *n* (%)	462 (68.0%)	105 (71.9%)	97 (66.9%)	0.598
BMI, kg/m2	26.6 ± 3.4	26.3 ± 3.6	26.7 ± 3.4	0.599
WHR, %	87.6 ± 6.0	87.5 ± 6.8	87.6 ± 5.9	0.974
Current smoker, *n* (%)	174 (25.6%)	28 (19.2%)	30 (20.7%)	0.156
**Office BP parameters**				
Office SBP, mmHg	126.5 ± 14.5	125.0 ± 15.5	125.3 ± 13.5	0.403
Office DBP, mmHg	85.2 ± 11.5	83.5 ± 13.2	85.3 ± 11.3	0.272
Office MAP, mmHg	99.0 ± 11.7	97.3 ± 13.1	98.6 ± 11.4	0.327
Office PP, mmHg	41.4 ± 9.9	41.5 ± 10.5	40.0 ± 8.6	0.295
**Antihypertensive drug usage**				
Antihypertensive drug number, *n*	1.5 ± 1.0	1.6 ± 1.0	1.4 ± 0.9	0.250
ACEI/ARB, *n* (%)	279 (41.1%)	74 (50.7%)	57 (39.3%)	0.076
Beta-blocker, *n* (%)	308 (45.4%)	69 (47.3%)	60 (41.4%)	0.576
CCB, *n* (%)	286 (42.1%)	56 (38.4%)	63 (43.4%)	0.637
Thiazide, *n* (%)	105 (15.5%)	31 (21.2%)	21 (14.5%)	0.191
Spironolactone, *n* (%)	3 (0.4%)	2 (1.4%)	2 (1.4%)	0.290
Alpha-blocker, *n* (%)	16 (2.4%)	2 (1.4%)	4 (2.8%)	0.700
Combination of ACEI/ARB and CCB, *n* (%)	48 (7.1%)	13 (8.9%)	10 (6.9%)	0.726
Combination of ACEI/ARB and thiazide, *n* (%)	26 (3.8%)	7 (4.8%)	3 (2.1%)	0.449
Combination of CCB and thiazide, *n* (%)	9 (1.3%)	3 (2.1%)	1 (0.7%)	0.598
Combination of ACEI/ARB and CCB and thiazide, *n* (%)	21 (3.1%)	1 (0.7%)	4 (2.8%)	0.263
Combination of ACEI/ARB and beta-blocker and CCB and thiazide, *n* (%)	10 (1.5%)	3 (2.1%)	0 (0.0%)	0.269
**Biochemical profiles**				
TC, mg/dL	194.5 ± 34.7	200.4 ± 36.0	197.0 ± 37.7	0.171
TG, mg/dL	168.2 ± 110.1	172.3 ± 135.0	150.0 ± 94.7	0.158
HDL-C, mg/dL	44.8 ± 11.1	46.2 ± 13.9	47.4 ± 14.0	0.038
LDL-C, mg/dL	125.1 ± 31.4	128.9 ± 31.5	127.2 ± 31.7	0.359
Creatinine, mg/dL	0.8 ± 0.2	0.9 ± 0.2	0.8 ± 0.2	0.553
eGFR, mL/min/1.73 m2	130.0 ± 38.1	126.4 ± 41.0	129.4 ± 36.5	0.594
Sodium, mmol/L	141.2 ± 2.6	141.6 ± 2.8	141.2 ± 2.4	0.273
Potassium, mmol/L	4.1 ± 0.3	4.0 ± 0.3	4.0 ± 0.3	0.421
ALT, U/L	27.8 ± 19.2	27.2 ± 18.9	26.8 ± 20.0	0.805
UA, mg/dL	6.7 ± 1.7	6.7 ± 1.8	6.5 ± 1.6	0.421
Glucose, mg/dL	98.1 ± 9.1	97.5 ± 9.0	98.4 ± 10.0	0.667
Aldosterone, pg/mL	225.4 ± 126.9	232.6 ± 97.0	225.1 ± 115.2	0.799
**Ambulatory BP parameters**				
24-h SBP, mmHg	123.1 ± 12.1	123.0 ± 12.7	124.1 ± 11.8	0.659
24-h DBP, mmHg	82.6 ± 9.4	81.7 ± 10.0	83.0 ± 9.6	0.483
Daytime SBP, mmHg	125.9 ± 12.7	126.1 ± 13.3	126.9 ± 12.3	0.670
Daytime DBP, mmHg	85.0 ± 9.8	84.3 ± 10.4	85.2 ± 9.9	0.661
Nighttime SBP, mmHg	114.1 ± 12.7	113.0 ± 13.1	114.5 ± 12.7	0.568
Nighttime DBP, mmHg	74.9 ± 9.8	73.5 ± 10.4	75.4 ± 10.2	0.219
MH/MUCH, *n* (%)	264 (38.9%)	64 (43.8%)	58 (40.0%)	0.539

*ACEI, angiotensin converting enzyme inhibitor; ALT, alanine aminotransferase; ANN, artificial neural networks; ARB, angiotensin receptor blocker; BP, blood pressure; BMI, body mass index; CCB, calcium channel blocker; DBP, diastolic blood pressure; eGFR, estimated glomerular filtration rate; HDL-C, high-density lipoprotein-cholesterol; LDL-C, low-density lipoprotein-cholesterol; LR, logistic regression; MAP, mean arterial pressure; MH, masked hypertension; MUCH, masked uncontrolled hypertension; PP, pulse pressure; RF, random forest; SBP, systolic blood pressure; TC, total cholesterol; TG, triglyceride; UA, uric acid; WHR, waist-hip ratio; XGboost, eXtreme Gradient Boosting*.

### Proportion of MH/MUCH

In cohort 1, 386 patients fulfilled the criteria of MH/MUCH (39.8%). In cohort 2, 140 patients fulfilled the criteria of MH/MUCH (33.7%). The proportions of MH/MUCH were higher in cohort 1 (*P* = 0.031) (Table 1).

The proportions of MH/MUCH were similar among the training set (38.9%), validation set (43.8%), and test set (40.0%) (Table 2).

### Hyperparameters and Importance Rank of Candidate Variables

The tuned hyperparameters in the four models are presented in Supplementary Table 3. The top 10 important variables and their importance in each model are listed in Supplementary Table 4. The importance matrix plot of the RF model is presented in Supplementary Figure 2.

### Feature Selection

**Figure 2:**
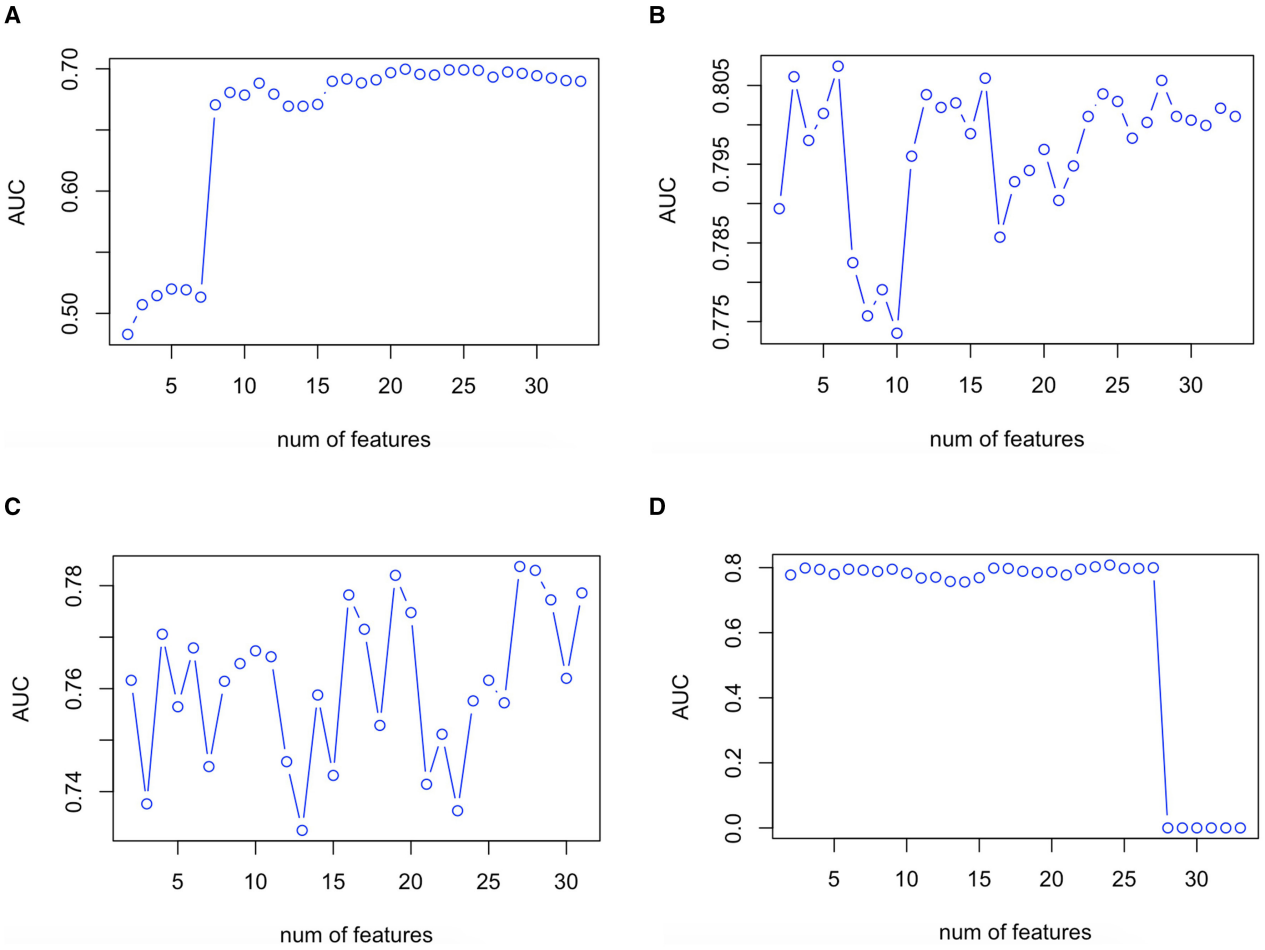
Validation plot of feature selection in the LR **(A)**, RF **(B)**, XGboost **(C)**, and ANN **(D)** models. First spot starts in two features. ANN, artificial neural network; AUC, area under the receiver operating characteristic curve; LR, logistic regression; RF, random forest; XGboost, eXtreme Gradient Boosting.

The validation plot showing the trend of AUC to the number of features in the validation set is shown in Figure 2. Twenty one predictor variables (spironolactone, alpha-blocker, beta-blocker, ACEI/ARB, male sex, current smoker, TC, office DBP, TG, LDL-C, office SBP, thiazide, CCB, office MAP, age, combination of ACEI/ARB and CCB, creatinine, combination of ACEI/ARB and thiazide, eGFR, HDL-C, and ALT, written in descending order of importance) obtained the largest AUC in the LR model. Six predictor variables (office DBP, office MAP, office SBP, office PP, beta-blocker, and HDL-C, written in descending order of importance) obtained the largest AUC in the RF model. Twenty seven predictor variables (office DBP, office SBP, potassium, office MAP, aldosterone, WHR, creatinine, TG, eGFR, HDL-C, LDL-C, office PP, BMI, glucose, sodium, age, TC, ALT, UA, dummy variable of taking beta-blocker, dummy variable of not taking CCB, antihypertensive drug number, dummy variable of not current smoker, male sex, dummy variable of not taking ACEI/ARB, dummy variable of not taking beta-blocker, and dummy variable of not taking thiazide, written in descending order of importance) obtained the largest AUC in the XGboost model. Twenty four predictor variables (office DBP, office MAP, eGFR, office SBP, spironolactone, combination of ACEI/ARB and beta-blocker and CCB and thiazide, current smoker, combination of ACEI/ARB and CCB and thiazide, TG, beta-blocker, age, office PP, thiazide, alpha-blocker, ALT, creatinine, combination of ACEI/ARB and CCB, WHR, male sex, HDL-C, BMI, sodium, combination of ACEI/ARB and thiazide, and aldosterone, written in descending order of importance) obtained the largest AUC in the ANN model (Figures 1, 2).

### Probability Threshold

The optimal probability thresholds were 0.211, 0.112, 0.020, and 0.245 for the LR, RF, XGboost, and ANN models, respectively.

### Performance of Prediction Models in Internal Validation

**Figure 3:**
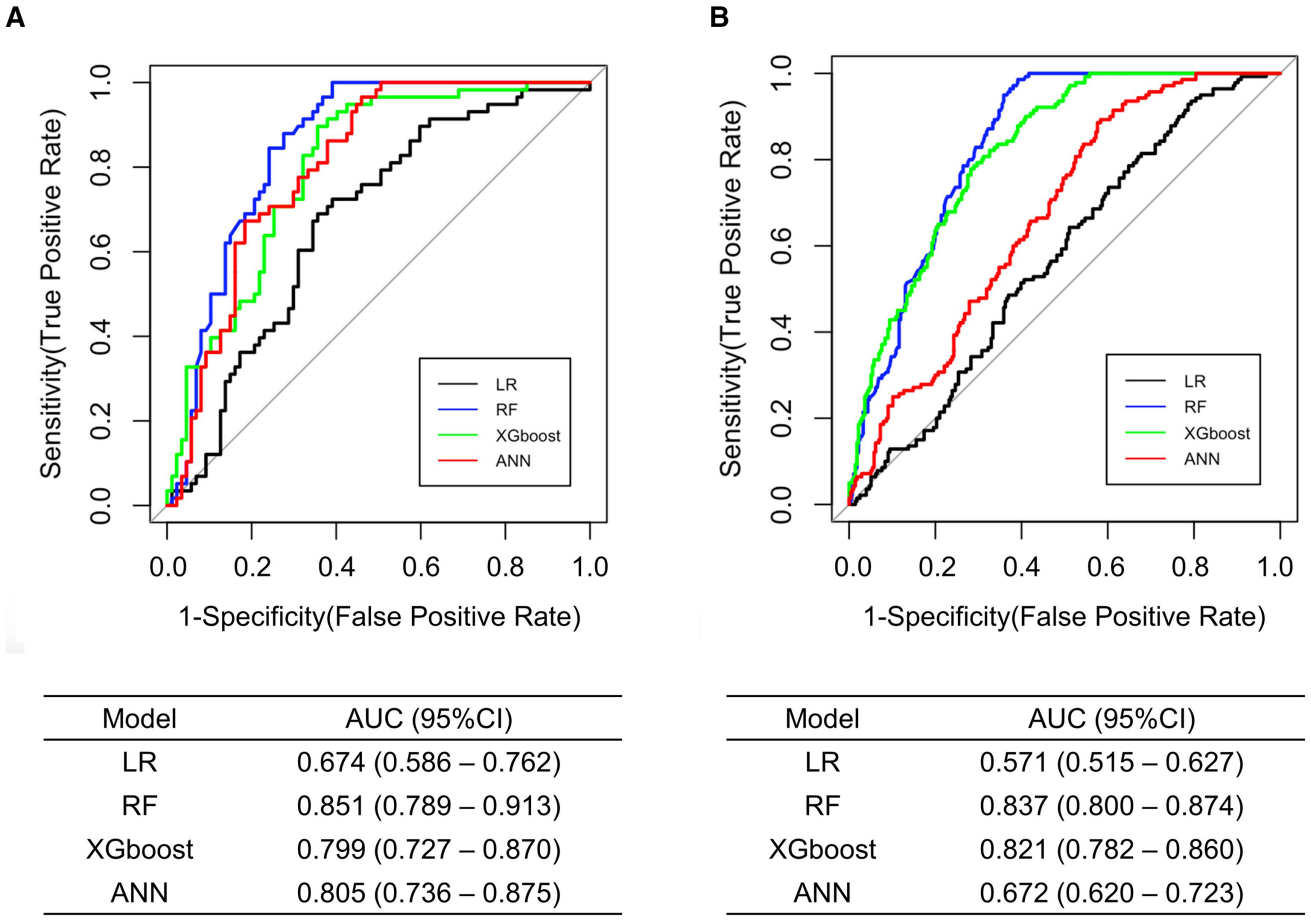
Comparison of the ROC curves and the AUC among the developed models for MH/MUCH prediction in internal **(A)** and external validation **(B)**. ANN, artificial neural network; AUC, area under the curve; CI, confidence interval; LR, logistic regression; MH/MUCH, masked hypertension/masked uncontrolled hypertension; RF, random forest; ROC, receiver operating characteristic; XGboost, eXtreme Gradient Boosting.

Figure 3A presents the ROC curves and the AUC obtained in the test set. The RF model exhibited the largest AUC (0.851, 95% CI 0.789–0.913), whereas the LR model exhibited the smallest AUC (0.674, 95% CI 0.586–0.762).

The confusion matrix and performance metrics of the four models in the test set were assessed (Table 3). All models had a high sensitivity for the prediction of MH/MUCH (0.914–1.000). The NPVs in the RF, XGboost, and ANN models were also high (0.927–1.000). However, the specificity (0.333–0.609), PPV (0.478–0.630), and accuracy (0.566–0.766) were relatively low in the four models. Among the four models, the RF model exhibited the highest sensitivity (1.000, 95% CI 1.000–1.000), specificity (0.609, 95% CI 0.507–0.712), PPV (0.630, 95% CI 0.532–0.729), NPV (1.000, 95% CI 1.000–1.000), accuracy (0.766, 95% CI 0.688–0.832), and F_1_ score (0.773).

**Table 3:** Confusion matrix and performance metrics among the developed models for MH/MUCH prediction (1 represents with MH/MUCH; 0 represents without MH/MUCH).

	**Model**			**Actual** **MH/MUCH**		**Sensitivity** **(95% CI)**	**Specificity** **(95% CI)**	**PPV** **(95% CI)**	**NPV** **(95% CI)**	**Accuracy** **(95% CI)**
				**1**	**0**					
**Internal validation**	LR	**Predicted MH/MUCH**	**1** **0**	53 5	58 29	0.914 (0.842–0.986)	0.333 (0.234–0.432)	0.478 (0.385–0.570)	0.853 (0.734–0.972)	0.566 (0.481–0.648)
	RF		**1** **0**	58 0	34 53	1.000 (1.000–1.000)	0.609 (0.507–0.712)	0.630 (0.532–0.729)	1.000 (1.000–1.000)	0.766 (0.688–0.832)
	XGboost		**1** **0**	54 4	36 51	0.931 (0.866–0.996)	0.586 (0.483–0.690)	0.600 (0.499–0.701)	0.927 (0.859–0.996)	0.724 (0.644–0.795)
	ANN		**1** **0**	55 3	39 48	0.948 (0.891–1.005)	0.552 (0.447–0.656)	0.585 (0.486–0.685)	0.941 (0.877–1.006)	0.710 (0.629–0.783)
**External validation**	LR	**Predicted MH/MUCH**	**1** **0**	133 7	227 49	0.950 (0.914–0.986)	0.178 (0.132–0.223)	0.369 (0.320–0.419)	0.875 (0.788–0.962)	0.438 (0.389–0.487)
	RF		**1** **0**	140 0	116 160	1.000 (1.000–1.000)	0.580 (0.521–0.638)	0.547 (0.486–0.608)	1.000 (1.000–1.000)	0.721 (0.675–0.764)
	XGboost		**1** **0**	137 3	149 127	0.979 (0.955–1.003)	0.460 (0.401–0.519)	0.479 (0.421–0.537)	0.977 (0.951–1.003)	0.635 (0.586–0.681)
	ANN		**1** **0**	138 2	213 63	0.986 (0.966–1.005)	0.228 (0.179–0.278)	0.393 (0.342–0.444)	0.969 (0.927–1.011)	0.483 (0.434–0.532)

*ANN, artificial neural network; CI, confidence interval; LR, logistic regression; MH/MUCH, masked hypertension/masked uncontrolled hypertension; NPV, negative predictive value; PPV, positive predictive value; RF, random forest; XGboost, eXtreme Gradient Boosting*.

Supplementary Figures 3, 4 present the example decision tree plots in the training set of the RF and XGboost models, respectively.

### Performance of Prediction Models in External Validation

The ROC curves and the AUC are shown in Figure 3B. Similar to the results in internal validation, the RF model exhibited the largest AUC (0.837, 95% CI 0.800–0.874), whereas the LR model exhibited the smallest AUC (0.571, 95% CI 0.515–0.627).

The confusion matrix and performance metrics of the four models in the external validation set were assessed (Table 3). All models had high sensitivity for the prediction of MH/MUCH (0.950–1.000). The NPVs in the RF, XGboost, and ANN models were also high (0.969–1.000). However, the specificity (0.178–0.580), PPV (0.369–0.547), and accuracy (0.438–0.721) were relatively low. Among the four models, the RF model exhibited the highest sensitivity (1.000, 95% CI 1.000–1.000), specificity (0.580, 95% CI 0.521–0.638), PPV (0.547, 95% CI 0.486–0.608), NPV (1.000, 95% CI 1.000–1.000), accuracy (0.721, 95% CI 0.675–0.764), and F_1_ score (0.707).

## Discussion

In the present study, we developed four models for MH/MUCH prediction using patient features obtained in a single outpatient visit and tested them. All models had high sensitivity and NPV. The RF, XGboost, and ANN models had AUC and F_1_ scores that surpassed those of the LR model. Among them, the RF model, composed of 6 predictor variables, exhibited the best overall performance. In addition, age, male sex, current smoker, office SBP, office DBP, office MAP, office PP, eGFR, creatinine, TG, HDL-C, ALT, beta-blocker, and thiazide were selected as predictor variables in more than three models, indicating their close association with MH/MUCH.

Patients with MH/MUCH had a significantly higher risk of cardiac/cerebrovascular events than those with controlled hypertension but a similar risk to those with sustained hypertension (9, 10). Identifying these patients and initiating appropriate treatment is a priority. Currently, out-of-office BP monitoring, either ABPM or HBPM, is the gold standard to diagnose these patients (4–7). However, the use of out-of-office BP monitoring is usually limited for many reasons, such as the shortage of resources, great consumption of time, poor compliance, and poor adherence of patients (31, 32). It is important to find a more efficient way to identify this particular patient group.

To the best of our knowledge, the present study is the first to report the development and evaluation of prediction models for MH/MUCH. The strength of the present study is the reasonable discrimination of the RF model in the external validation set, despite the high dissimilarity between cohort 1 and 2. The temporal, geographical, and domain validation of our model (33) prove its transportability and applicability to actual outpatient settings. It was suggested that a high NPV, as in the present study, is desirable when a condition is serious, largely asymptomatic, or if treatment for a condition is advisable early in its course (34), which matches the features of MH/MUCH (24).

The reason the RF model produced the best performance may be attributable to its ability to overcome the multicollinearity of our given data (35). The RF algorithm was previously used to define SBP variability features for cardiovascular outcome prediction in the Systolic Blood Pressure Intervention Trial (SPRINT) trial (36). While interpreting the importance of multi-colinear variables is still difficult in the RF algorithm, accuracy is much less affected (37), making it a favorable algorithm. Some of the given variables in our dataset are highly correlated (Supplementary Figure 1), creating a significant hindrance to linear algorithms such as LR (38).

It is interesting to point out that eGFR and creatinine were included as predictor variables in three models. Several studies have shown that MH/MUCH is associated with the development of chronic kidney disease (CKD) and the progression of kidney disease (16, 17). MUCH/MH is also common in patients with CKD and associated with lower eGFR (18), which is consistent with our finding that eGFR and creatinine were important variables for the prediction of MH/MUCH.

In the present study, HDL-C and TG were predictor variables selected in all and three models, respectively. Previous studies have found a correlation between metabolic syndrome and MH/MUCH (6, 19, 20). Although one study reported that MH patients had greater waist circumference and lower HDL-C than normotensives (19), another study showed that only office BP contributed significantly (20). Our results suggest that among the criteria for metabolic syndrome, HDL-C and TG have higher significance with the exception of office BP. These findings mark the complexity of MH/MUCH pathophysiology, and also imply that different parameters in metabolic syndrome have variable degrees of impact or association with MH/MUCH, providing us with further insights into the underlying mechanisms.

It has been suggested by previous studies that patients with MH/MUCH tend to have a more active sympathetic tone out of the office due to neurogenic abnormalities (21–23). In the present study, beta-blocker and alpha-blocker are chosen in all and two models, respectively, and these drugs are sympathetic antagonists commonly used to treat CVD and hypertension. However, these associations are indicated by cross-sectional comparisons, and direct causal inferences cannot be ascertained.

As for demographic variables, previous studies showed that smoking was associated with MH/MUCH (5, 6, 24–27). The prevalence of MH/MUCH is also found to be greater in men (5, 6, 24, 27). It is consistent with our finding that current smoker and male sex were important variables for the prediction of MH/MUCH.

In the present study, MH/MUCH was defined according to daytime as well as nighttime ambulatory BP. Patients with MH/MUCH increased from 276 (28.5%) to 386 (39.8%) in cohort 1 and increased from 70 (16.8%) to 140 (33.7%) in cohort 2 when we included nighttime BP as one of our criteria to define MH/MUCH aside from office BP, 24-h average BP, and daytime ambulatory BP. High prevalence of nighttime MUCH (or masked uncontrolled nocturnal hypertension) was also noted in the study by Coccina F et al. in which 357 (48.5%) patients among 738 treated hypertensive patients were reported to have nighttime MUCH (39). In their study, patients with nighttime MUCH had an increased risk of cardiovascular events compared to those with controlled hypertension. With our models, physicians could identify patients with not only daytime but also nighttime MH/MUCH.

### Study Limitations

The current study has several limitations that must be considered. First, external validation was only performed in patients with hypertension in Taiwan. Data with more representation of diverse populations, a larger sample size, and untreated patients must be obtained to demonstrate better transportability. Second, there were some differences between the inclusion criteria of the two cohorts. Compared to cohort 2, cohort 1 had additional inclusion criteria of “age ≤ 50 years old,” “BMI ≤ 35 kg/m^2^”, and “fast glucose level <126 mg/dL with no diabetes mellitus.” Despite the differences of baseline characteristics between the two cohorts, the performance of our models in external validation was acceptable. Third, HBPM was not included in the diagnostic criteria for MH/MUCH. Even though previous studies showed a greater importance of ABPM to MH (24, 40), the present study may be limited by not identifying all MH/MUCH patients. Forth, some variables found to be related to MH/MUCH were not available in our cohorts, such as echocardiographic variables (41). Finally, our models were developed to predict MH and MUCH together. However, there are potential different pathophysiology and etiology behind MH and MUCH (6, 24). Although some previous studies also did not differentiate MUCH from MH (41–44), further studies should be considered to develop models of MH and MUCH separately in order to increase the accuracy of prediction models.

## Conclusion

Patients with MH/MUCH are at an increased risk of CVD compared to patients with controlled hypertension. Due to their “masking nature,” they are, however, largely underdiagnosed and often left untreated. Our machine learning-based prediction models, especially RF, could assist physicians with their ability to detect MH/MUCH patients using clinical data obtained in a single outpatient visit. Through timely and proper handling of these models, patients with MH/MUCH could be able to receive early diagnosis and appropriate treatment to prevent cardiovascular events in the future.

## Data Availability Statement

The raw data supporting the conclusions of this article will be made available by the authors, without undue reservation.

## Ethics Statement

The studies involving human participants were reviewed and approved by Taipei Veterans General Hospital. The patients/participants provided their written informed consent to participate in this study.

## Author Contributions

M-HH, L-CS, and Y-CW contributed to conception and design, analysis and interpretation of data, and drafted the manuscript. H-BL, P-HH, T-CW, S-JL, W-HP, and J-WC contributed to data acquisition and drafted the manuscript. C-CH contributed to conception, data acquisition, analysis and interpretation of data, and drafted and critically revised the manuscript. All authors gave final approval and agreed to be accountable for all aspects of work ensuring integrity and accuracy.

## Funding

The authors disclosed receipt of the following financial support for the research, authorship, and/or publication of this article: This work was supported by research grants V101B-004, V102B-024, V103C-019, V104C-025, V106C-120, V108C-151, VGHUST108-G1-3-2, VTA108-V1-7-2, and V110C-058 from Taipei Veterans General Hospital, Taipei, Taiwan, ROC and by research grant MOST108-2314-B-075-062-MY3 from the Ministry of Science and Technology, Taiwan, ROC. The funders had no role in data collection or preparation of the manuscript.

## Conflict of Interest

The authors declare that the research was conducted in the absence of any commercial or financial relationships that could be construed as a potential conflict of interest.

## Publisher's Note

All claims expressed in this article are solely those of the authors and do not necessarily represent those of their affiliated organizations, or those of the publisher, the editors and the reviewers. Any product that may be evaluated in this article, or claim that may be made by its manufacturer, is not guaranteed or endorsed by the publisher.
