# Supplementary material for: Prediction of Masked Hypertension and Masked Uncontrolled Hypertension Using Machine Learning
Source: Front Cardiovasc Med. 2021 Nov 19;8:778306. doi: 10.3389/fcvm.2021.778306 (PMC8639874; doi:10.3389/fcvm.2021.778306)
Supplement: Supplementary file 1 [file Data_Sheet_1.pdf]

Supplementary Material

1 Supplementary Material

Supplementary Material 1. Data collection protocol

History taking and physical checkup

All patients were evaluated at the hypertensive clinics. Comprehensive history taking and physical checkup were conducted by a hypertensive specialist. Body weight and height were recorded in patients without shoes and wearing only light indoor clothes. Body mass index was defined as weight in kilograms divided by the square of height in meters. Waist circumference was measured midway between the iliac crest and the lower margin of the ribs. The hip circumference was measured at the maximum circumference of the buttocks, with the subject standing with feet placed together. The waist-to-hip ratio was calculated as 100 × (waist circumference in centimeters/hip circumference in centimeters). Drug use was also recorded.

BP measurement

Office blood pressure (BP) was measured according to a standardized protocol by a well-trained nurse with an electronic BP monitor (cohort 1, Omega 1400 NBP, Invivo Research Inc, Orlando, FL, USA; cohort 2, Omron HEM-7121, Omron Healthcare Taiwan Co., Songshan, Taipei, Taiwan, ROC) in the morning hours after patients had been instructed to sit for 10 min in a quiet room. Three consecutive BP measurements were obtained from the same upper arm. Each measurement was taken at 30-s intervals. Patients were connected to the ambulatory BP monitoring device (cohort 1, model 90207, SpaceLabs, Redmond, Washington, USA; cohort 2, WatchBP O3 ambulatory blood pressure monitor, Microlife Corp., Neihu, Taipei, Taiwan, ROC). In cohort 1, the device was programmed to record BP every 20 min from 0600h until 2300h and 30 min from 2300h to 0600h. In cohort 2, the device was programmed to record BP every 15 min between 0600h and 2200h and every 60 min from 2200h to 0600h.

Laboratory measurements

Blood samples were collected in the morning after overnight fasting. The fasting whole blood samples of the participants were collected via venipuncture after the participants rested for 10 min while in the supine position. The participants were instructed to take all routine medications as they normally would. All blood samples were sent to the central laboratory for analysis. Serum levels of total cholesterol, triglyceride, high-density lipoprotein cholesterol, low-density lipoprotein cholesterol, blood urea nitrogen, creatinine, sodium, potassium, aspartate aminotransferase, alanine aminotransferase, uric acid, fasting blood glucose, and insulin were also measured. Insulin resistance was calculated using a homeostasis model assessment for insulin resistance (1). Serum aldosterone and plasma renin activity were determined (2). The baseline estimated glomerular filtration rate was calculated using the four-variable equation proposed by the Modification of Diet in Renal Disease Study.

24-hour urine collection

Patients in cohort 1 underwent 24-hour urine collection. Each participant was provided with a urine sample container. Urine samples were collected over a 24-hour period by a well-trained nurse. Complete oral and written guidance about urine specimen collection, transportation, and preservation were also provided. On the day of urine collection, they were required to follow their daily diet habits and advised to avoid strenuous exercise to reduce sweating. They were required to discard the first voided urine upon waking up in the morning and to collect all voided urine during the subsequent 24 hours, including the first void sample of the following morning. Upon completion of collection, a well-trained nurse recorded the urine volume in each collection container to determine the total urine volume during the 24-hour collection period. All samples of urine were sent to a central laboratory to determine levels of urinary sodium, potassium, chloride, creatinine, and total protein. Urinary norepinephrine, epinephrine, and dopamine were measured with high-pressure liquid chromatography-electrochemical detection (3).

Supplementary Material 2. Details of feature selection

Logistic regression (LR)

LR model accurately predicts the probability of the binary dependent variable using the maximum likelihood estimation to determine the regression coefficient. Feature selection was performed in following steps: (a) combine train and validation set as dataset X; (b) fit dataset X with the LR model; (c) take the absolute value of LR coefficient β; (d) perform stepwise LR with backward step Akaike information criterion (stepAIC) feature selection (forward stepAIC and both stepAIC feature selection were also performed in our study, but with poorer performance) (4).

Random forest (RF)

RF model is a bagging ensemble method that averages the results over multiple decision trees from randomly selected subsamples through majority voting (5). Feature selection was performed in following steps: (a) combine train and validation set as dataset X; (b) use Boruta package, one of the wrappers built around the RF classification algorithm to get the importance, which is based on mean decrease accuracy (6); (c) rank feature importance and to draw the importance matrix plot.

eXtreme Gradient Boosting (XGboost)

XGboost is an optimized distributed gradient boosting library that provides superior prediction through the conversion of a set of weak learners to strong learners (7). Feature selection was performed in following steps: (a) combine train and validation set as dataset X; (b) fit dataset X with the XGBoost model; (c) get relative contribution of the corresponding feature to the XGBoost model by taking each feature's contribution for each tree in the XGBoost model as importance. However, the exact computation of the importance is undocumented in getFeatureImportance {mlr}.

Artificial neural network (ANN)

The deep neural network is a multilayer perceptron with multiple hidden layers optimized using stochastic gradient descent. Rectified linear unit activation was used for all hidden layers. Feature selection was performed in following steps: (a) combine train and validation set as dataset X; (b) fit dataset X with the ANN model; (c) use package h2o built-in variable importance as importance, which is based on Gedeon method (This implementation considers the weights connecting the input features to the first two hidden layers and provides, for each features, the relative importance normalized between 0 and 1) (8).

After getting importance of each model, we sorted features based on importance and added each feature one-by-one into model. We calculated AUC of validation set each time we added new feature, and chose the feature number with the maximum AUC.

Supplementary Material 3. Probability threshold

In order to maximize sensitivity and reduce type II error, we initially implemented F_2 score as the target of threshold-moving since F_2 score weighs sensitivity more than F_1 score (9). However, after testing both F_1 and F_2 score as the target of threshold-moving, our results suggested that using F_2 score could indeed improve sensitivity, but at too much expense of specificity and PPV. Thus, in order to make it a more balanced model, we decided to use F_1 score while selecting decision threshold.

References of supplementary material

1. Rudenski AS, Matthews DR, Levy JC, et al. Understanding "insulin resistance": both glucose resistance and insulin resistance are required to model human diabetes. Metabolism (1991) 40: 908-917. doi: 10.1016/0026-0495(91)90065-5

2. Sealey JE. Plasma renin activity and plasma prorenin assays. Clin Chem (1991) 37: 1811-1819.

3. Peitzsch M, Pelzel D, Glöckner S, et al. Simultaneous liquid chromatography tandem mass spectrometric determination of urinary free metanephrines and catecholamines, with comparisons of free and deconjugated metabolites. Clin Chim Acta (2013) 418: 50-58. doi: 10.1016/j.cca.2012.12.031

4. McElreath R. Statistical Rethinking: A Bayesian Course with Examples in R and STAN. 2nd ed. Boca Raton: CRC Press (2020).

5. Thomas C. Data Mining. London: IntechOpen (2018).

6. Kursa MB, Rudnicki WR. Feature selection with the Boruta package. J Stat Softw (2010) 36: 1-13. doi: 10.1371/journal.pone.0107801

7. Chen T, Guestrin C. XGBoost: A scalable tree boosting system. In: proceedings of the 22nd ACM SIGKDD International Conference on Knowledge Discovery and Data Mining (2016) San Francisco, CA. p.785-794.

8. Gedeon TD. Data mining of inputs: analysing magnitude and functional measures. Int J Neural Syst (1997) 8(2):209-18. doi: 10.1142/s0129065797000227

9. Wotawa F, Friedrich G, Pill I, Koitz-Hristov R, Moonis A. Advances and trends in artificial intelligence. From theory to practice. In: proceedings of 32nd International Conference on Industrial, Engineering and Other Applications of Applied Intelligent Systems, IEA/AIE (2019) Graz, Austria. p.343-344.

2 Supplementary Figures

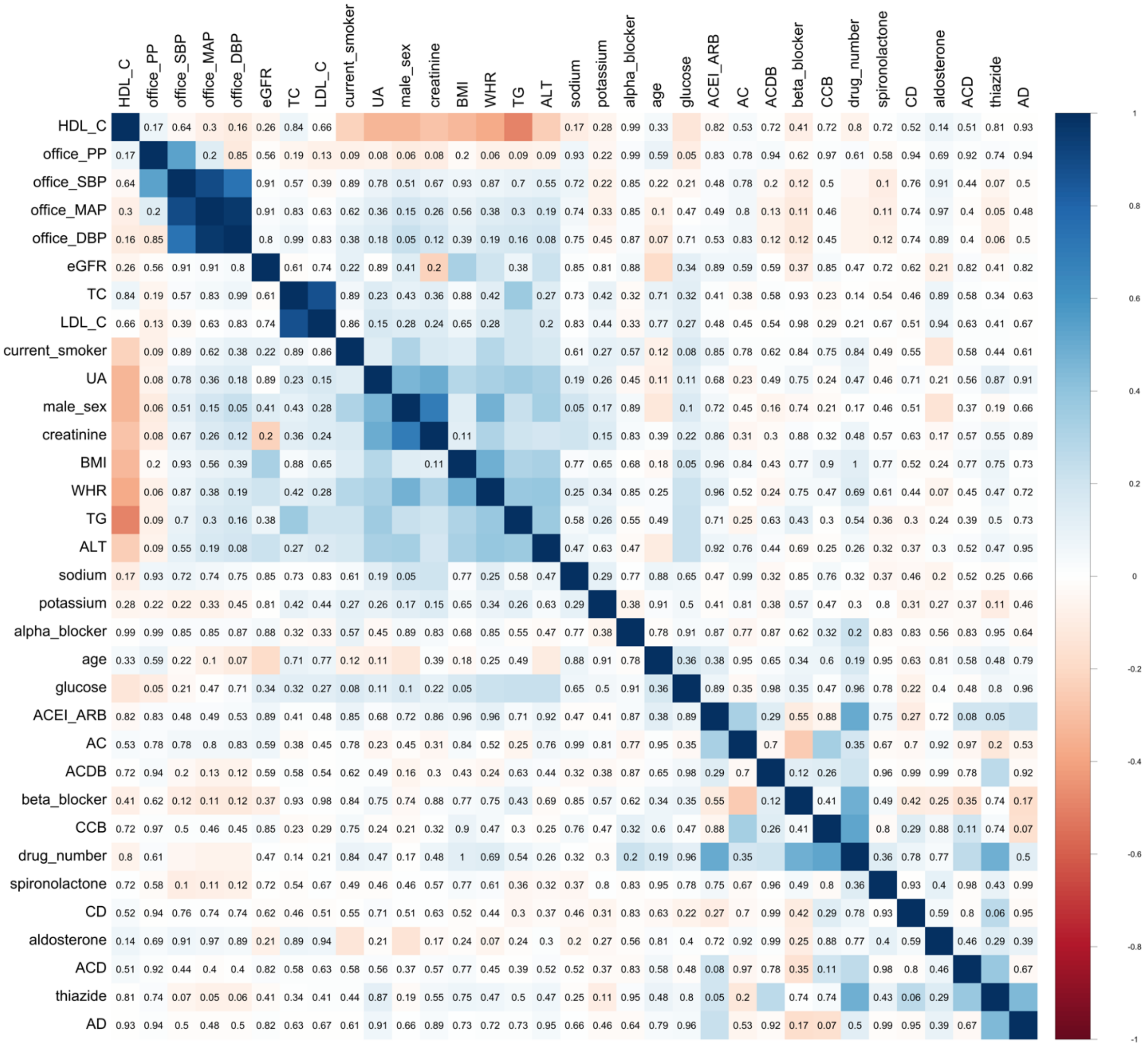

Supplementary Figure 1. Heatmap of the Spearman's correlation coefficients (the colors of squares) with significance levels (the numbers within squares).

ACEI_ARB, angiotensin-converting enzyme inhibitor/angiotensin receptor; ACD, combination of ACEI/ARB and CCB and thiazide; AC, combination of ACEI/ARB and CCB; ACDB, combination of ACEI/ARB and CCB and thiazide and beta-blocker; AD, combination of ACEI/ARB and thiazide; ALT, alanine aminotransferase; BMI, body mass index; CCB, calcium channel blocker; CD, combination of CCB and thiazide; DBP, diastolic blood pressure; eGFR, estimated glomerular filtration rate; HDL_C, high-density lipoprotein-cholesterol; LDL_C, low-density lipoprotein-cholesterol; MAP, mean arterial pressure; PP, pulse pressure; SBP, systolic blood pressure; TC, total cholesterol; TG, triglyceride; UA, uric acid; WHR, waist-to-hip ratio.

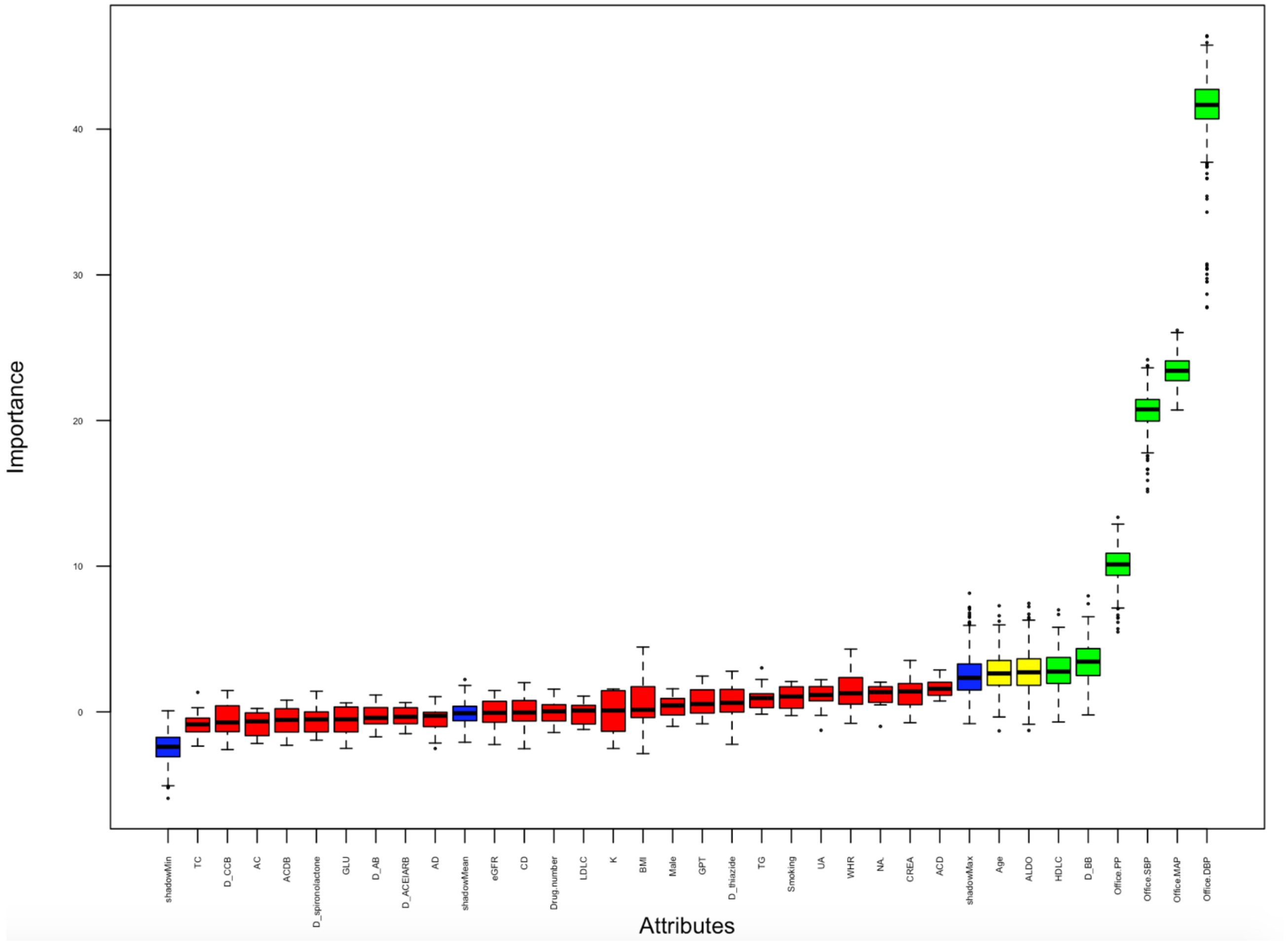

Supplementary Figure 2. Importance matrix plot of the RF model

ACD, combination of ACEI/ARB and CCB and thiazide; AC, ACEI/ARB and CCB; ACDB, combination of ACEI/ARB and CCB and thiazide and beta-blocker; AD, combination of ACEI/ARB and thiazide; ALDO, aldosterone; BMI, body mass index; CD, combination of CCB and thiazide; CREA, creatinine; D_AB, alpha blocker; D_ACEI/ARB, angiotensin-converting enzyme inhibitor/angiotensin receptor; D_BB, beta-blocker; DBP, diastolic blood pressure; D_CCB, calcium channel blocker; D_spironolactone, spironolactone; D_thiazide, thiazide; eGFR, estimated glomerular filtration rate; GLU, glucose; GPT, alanine aminotransferase; HDLC, high-density lipoprotein-cholesterol; K, potassium; LDLC, low-density lipoprotein-cholesterol; MAP, mean arterial pressure; NA., sodium; PP, pulse pressure; SBP, systolic blood pressure; TC, total cholesterol; TG, triglyceride; UA, uric acid; WHR, waist-to-hip ratio.

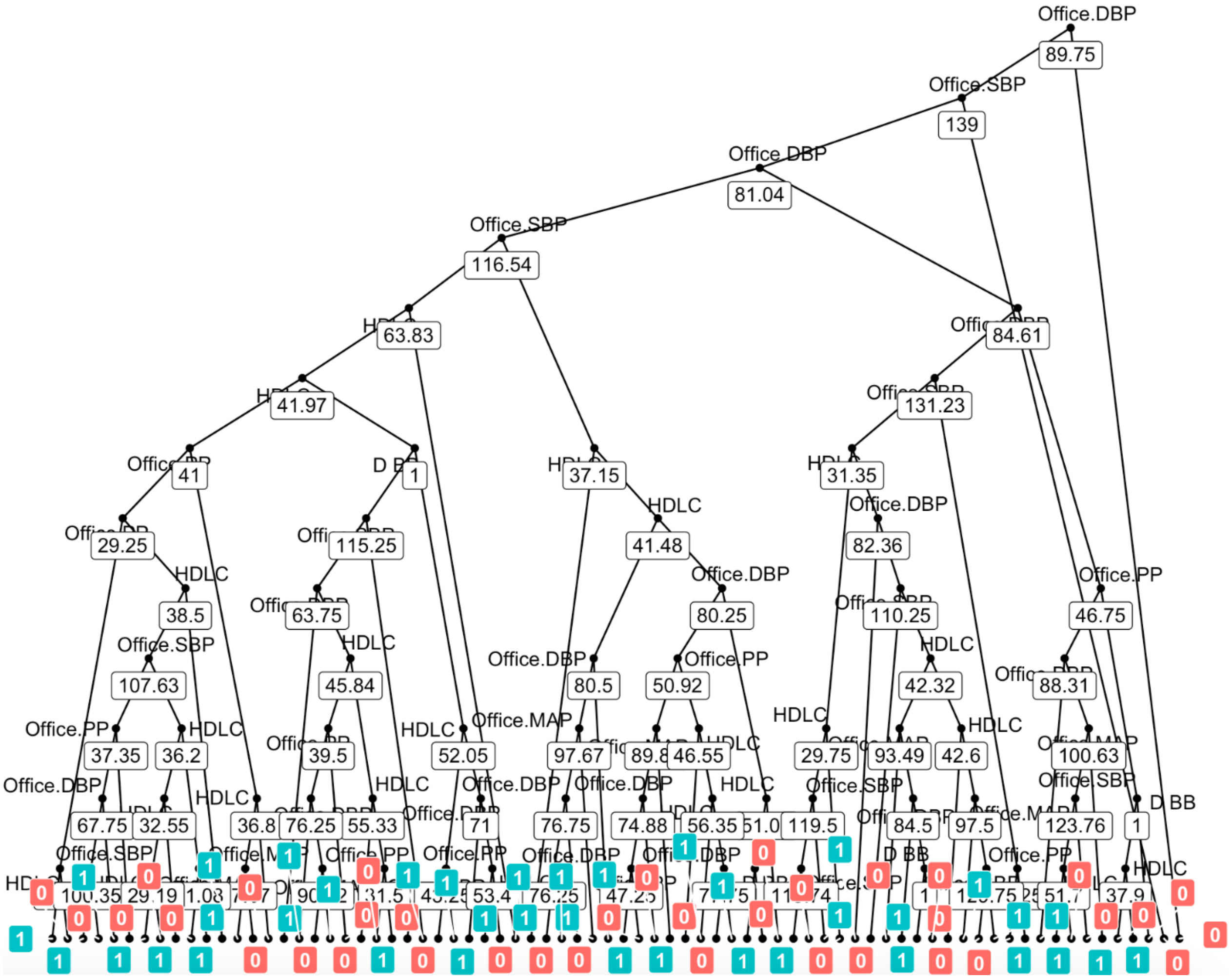

Supplementary Figure 3. Example decision tree plot of the RF model illustrating the classification of patients with (class = 1) and without (class = 0) MH/MUCH.

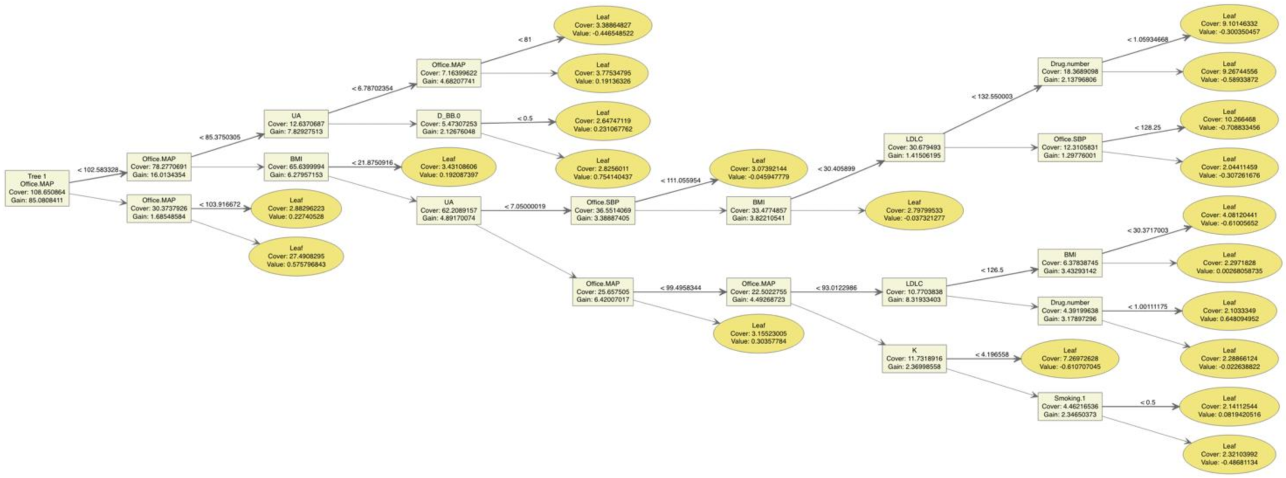

Supplementary Figure 4. Example decision tree plot of the XGboost model. Each box has the following components: selected variables for classification, value ($= \log(p/1-p)$), cover (impurity), and gain (loss of impurity).

3 Supplementary Tables

Supplementary Table 1. Complete variables in cohorts 1 and 2

	Cohort 1 (73 variables)	Cohort 2 (53 variables)
Demographic data	age, male sex, current smoker, drinking, height, weight, BMI, waist circumference, hip circumference, WHR, and wrist circumference	age, male sex, current smoker, height, weight, BMI, waist circumference, hip circumference, and WHR
Blood pressure parameters	office SBP, office DBP, office MAP, office PP, 24-hour SBP, 24-hour DBP, daytime SBP, daytime DBP, nighttime SBP, and nighttime DBP	office SBP, office DBP, office heart rate, office MAP, office PP, 24-hour SBP, 24-hour DBP, daytime SBP, daytime DBP, nighttime SBP, and nighttime DBP
Medication usage	ACEI/ARB, beta-blocker, CCB, thiazide, spironolactone, alpha-blocker, combination of ACEI/ARB and CCB, combination of ACEI/ARB and thiazide, combination of ACEI/ARB and CCB and thiazide, combination of CCB and thiazide, combination of ACEI/ARB and beta-blocker and CCB and thiazide, and antihypertensive drug number	renin inhibitor, ACEI/ARB, beta-blocker, CCB, thiazide, spironolactone, alpha-blocker, furosemide, anti-platelet drug, anti-coagulation drug, nitrite, lipid lowering drug, UA lowering drug, anti-diabetes drug, insulin, combination of ACEI/ARB and CCB, combination of ACEI/ARB and thiazide, combination of ACEI/ARB and CCB and thiazide, combination of CCB and thiazide, combination of ACEI/ARB and beta-blocker and CCB and thiazide, and antihypertensive drug number
Hemogram	white blood cell counts, red blood cell counts, hemoglobin, hematocrit, mean corpuscular volume, mean corpuscular hemoglobin, mean corpuscular hemoglobin concentration, and platelet counts	-
Biochemical profiles	high sensitivity CRP, glucose, TC, TG, low LDL-C, HDL-C, blood urea nitrogen, creatinine, eGFR, sodium, potassium, chloride, aspartate aminotransferase, ALT, UA, insulin, intact parathyroid hormone, thyroxine, plasma renin activity, insulin resistance, and aldosterone	TC, TG, HDL-C, LDL-C, creatinine, eGFR, sodium, potassium, ALT, UA, glucose, and aldosterone

24-hour urine profiles	total urine volume, sodium, potassium, chloride, creatinine, total protein, epinephrine, norepinephrine, dopamine, the summation of epinephrine and norepinephrine, and total catecholamine (the summation of epinephrine, norepinephrine, and dopamine)	-

ACEI, angiotensin converting enzyme inhibitor; ALT, alanine aminotransferase; ARB, angiotensin receptor blocker; BMI, body mass index; CCB, calcium channel blocker; DBP, diastolic blood pressure; eGFR, estimated glomerular filtration rate; HDL-C, high-density lipoprotein-cholesterol; LDL-C, low-density lipoprotein-cholesterol; MAP, mean arterial pressure; PP, pulse pressure; SBP, systolic blood pressure; TC, total cholesterol; TG, triglyceride; UA, uric acid; WHR, waist-hip ratio.

Supplementary Table 2. Algorithms and packages in RStudio

Algorithms and packages	Version	Identifier
Boruta	7.0.0	https://gitlab.com/mbq/Boruta/
caret	6.0-86	https://github.com/topepo/caret/`
corrplot	0.90	https://github.com/taiyun/corrplot
dataPreparation	1.0.1	https://CRAN.R-project.org/package=dataPreparation
dplyr	1.0.5	https://dplyr.tidyverse.org/
h2o	3.32.0.1	https://github.com/h2oai/h2o-3
magrittr	2.0.1	https://magrittr.tidyverse.org
MASS	7.3-53	http://www.stats.ox.ac.uk/pub/MASS4/
mlr3	0.11.0	https://mlr3.mlr-org.com/
pROC	1.17.0.1	https://www.expasy.org/resources/proc
randomForest	4.6-14	https://www.stat.berkeley.edu/~breiman/RandomForests/
rms	6.2-0	https://hbiostat.org/R/rms/
RSBID	0.0.0.9000	https://rdrr.io/github/dongyuanwu/RSBID/
tidymodels	0.1.3	https://tidymodels.tidymodels.org/
tidyverse	1.3.0	https://tidyverse.tidyverse.org/

Supplementary Table 3. The hyperparameters tuned in the four models

LR		RF	
Hyperparameter	Number	Hyperparameter	Number
lambda	0.001	mtry	15
		ntree	500
XGboost		ANN	
Hyperparameter	Number	Hyperparameter	Number
nrounds	120	epochs	100
eta	0.405	epsilon	1E-10
gamma	0.0537	hidden	[200,200]
lambda	0.209	l1	0.01
max_depth	8	l2	1.0E-4
min_child_weight	2	rho	0.999
subsample	0.593		
colsample_bytree	0.674		

ANN, artificial neural network; LR, logistic regression; RF, random forest; XGboost, eXtreme Gradient Boosting

Supplementary Table 4. The top 10 important variables in each model (absolute values of regression coefficients were written in parentheses for LR; importance was written in parentheses for RF, XGboost, and ANN)

Rank	LR	RF	XGboost	ANN
1	Spironolactone (0.901)	Office DBP (41.392)	Office DBP (0.213)	Office DBP (1.000)
2	Alpha-blocker (0.892)	Office MAP (23.427)	Office SBP (0.093)	Office MAP (0.798)
3	Beta-blocker (0.775)	Office SBP (20.701)	Potassium (0.066)	eGFR (0.654)
4	ACEI/ARB (0.669)	Office PP (10.096)	Office MAP (0.057)	Office SBP (0.586)
5	Male sex (0.516)	Beta-blocker (3.454)	Aldosterone (0.052)	Spironolactone (0.501)
6	Current smoker (0.424)	HDL-C (2.849)	WHR (0.045)	Combination of ACEI/ARB and beta-blocker and CCB and thiazide (0.499)
7	TC (0.416)	Aldosterone (2.737)	Creatinine (0.042)	Current smoker (0.483)
8	Office DBP (0.385)	Age (2.687)	TG (0.041)	Combination of ACEI/ARB and CCB and thiazide (0.435)
9	TG (0.361)	Combination of ACEI/ARB and CCB and thiazide (1.615)	eGFR (0.037)	TG (0.422)
10	LDL-C (0.359)	WHR (1.443)	HDL-C (0.036)	Beta-blocker (0.414)

ACEI/ARB, angiotensin-converting enzyme inhibitor/angiotensin receptor blocker; ANN, artificial neural network; CCB, calcium channel blocker; DBP, diastolic blood pressure; eGFR, estimated glomerular filtration rate; HDL-C, high-density lipoprotein-cholesterol; LDL-C, low-density lipoprotein-cholesterol; LR, logistic regression; MAP, mean arterial pressure; PP, pulse pressure; RF, random forest; SBP, systolic blood pressure; TC, total cholesterol; TG, triglyceride; WHR, waist-to-hip ratio; XGboost, eXtreme Gradient Boosting.
